# Ultrasound can assess soft tissue laxity in osteoarthritic knees and after total knee arthroplasty

**DOI:** 10.1002/jeo2.70461

**Published:** 2025-10-09

**Authors:** Ammar Ghabi, Bernard de Geofroy, Lilia Gharbi, Christopher Perlak, Robin Hattenberger, Jérôme Desmolliens, Jean‐François Gonzalez, Grégoire Micicoi

**Affiliations:** ^1^ Orthopaedic Surgery and Traumatology Department Military Hospital Laveran Marseille France; ^2^ University Institute of Locomotor and Sports (iULS), Pasteur II Hospital Nice France; ^3^ ICARE Unit, Inserm U1091 Côte d'Azur University Nice France; ^4^ Radiology Department Military Hospital Laveran Marseille France

**Keywords:** knee laxity, osteoarthritis, stress radiography, total knee arthroplasty, ultrasound

## Abstract

**Purpose:**

Peripheral knee laxity is commonly assessed through clinical examination and stress radiographs, both before and after total knee arthroplasty (TKA). This study aimed to evaluate the efficacy of ultrasound in assessing peripheral knee laxity compared to radiographic stress imaging.

**Methods:**

This retrospective bicentric study included 68 patients (mean age: 71.2 ± 5.8 years; body mass index [BMI]: 28.7 ± 7), with 32 osteoarthritic knees (47.1%) and 36 post‐TKA knees (52.9%). Laxity was measured in millimetres using manual stress manoeuvres applied during both ultrasound and radiographic examinations at 0° and 30° of flexion, under varus and valgus conditions.

**Results:**

Under ultrasound, the unstressed medial joint space measured 10.6 ± 4.5 mm at 0° and 10.9 ± 4.2 mm at 30°; under valgus stress, it increased to 13.0 ± 4.9 and 14.0 ± 5.1 mm, respectively. The unstressed lateral joint space measured 11.8 ± 4.5 mm at 0° and 11.6 ± 4.0 mm at 30°, increasing to 14.8 ± 5.1 and 15.5 ± 5.9 mm under varus stress. No statistically significant differences were observed between ultrasound and radiographic measurements of lateral laxity (2.9 ± 2.3 vs. 2.96 ± 1.74 mm, Δ = 0.04 mm, *p* = 0.91) or medial laxity (2.35 ± 2.16 vs. 2.44 ± 1.55 mm, Δ = 0.1 mm, *p* = 0.78). Correlation coefficients between the modalities ranged from 0.5 to 0.7 (*p* < 0.001). At 30° of flexion, lateral laxity was significantly greater in the TKA group (4.9 vs. 2.8 mm, *p* < 0.01).

**Conclusion:**

Stress ultrasound is a reliable tool for analysing medial and lateral peripheral knee laxities in both osteoarthritic knees and after total knee arthroplasty. While it could requires trained personnel, this technique represents a viable alternative to conventional stress radiographs.

**Level of Evidence:**

Level II.

**Abbreviations**
ACL: anterior cruciate ligament
BMI: body mass index
CI: confidence interval
IRB: Institutional Review Board
MCL: medial collateral ligament
OA: osteoarthritis
TELOS: stress device used in radiographic evaluations
THA: total hip arthroplasty
TKA: total knee arthroplasty

## INTRODUCTION

Functional outcomes following total knee arthroplasty (TKA) remain inferior to those achieved after total hip arthroplasty (THA) [4]. This discrepancy is multifactorial but is partly explained by the frequent alteration of native knee kinematics after TKA [5, 7], particularly due to suboptimal ligament balancing during surgery [24]. Achieving ideal ligament balance can be challenging [1], and some studies estimate that 20%–30% of TKA patients will continue to experience chronic postoperative pain [6].

Preoperative evaluation of peripheral coronal ligament laxities is therefore essential in osteoarthritic patients, as it provides information on the severity of osteoarthritis, the reducibility of major deformities and the patient's physiological laxities. Postoperatively, such evaluation remains critical since residual instability may manifest as chronic pain and potentially lead to revision surgery [12, 24, 25]. Peripheral knee laxities are typically assessed clinically and through stress radiographs, both before and after TKA [8, 11, 26].

Ultrasound offers the advantage of being increasingly accessible during consultation, without exposing patients to radiation, and for some surgeons, it is a natural extension of the clinical exam. Furthermore, ultrasound has recently been shown to outperform magnetic resonance imaging in quantifying meniscal extrusion in osteoarthritic knees, and cross‐correlation‐based bone tracking now enables dynamic in‐clinic measurements of knee kinematics [3, 23].

To date, no study has evaluated the utility of ultrasound as a tool to analyse peripheral knee ligament laxities before and after TKA. This modality presents a compelling alternative, especially for surgeons who lack access to radiographic stress testing in their institutions, while ultrasound machines are becoming more widely available.

The hypothesis of this study was that ultrasound could provide differential measurements of medial and lateral laxities under varus and valgus stress, comparable to conventional radiographic techniques. The objective was to assess the efficacy of ultrasound in evaluating peripheral knee laxities compared to traditional radiographic stress testing with and without manual stress.

## METHODS

### Patient selection

Patients were included if they were over 18 years of age and presented either with bi‐ or tri‐compartmental knee osteoarthritis or had undergone posterior‐stabilised TKA. Exclusion criteria included: osteoarthritis due to instability, patients with constrained or hinged TKA implants (e.g., CCK‐type), and patients who declined participation.

A total of 68 patients were included: 32 knees (47%) with osteoarthritis and 36 knees (53%) post‐TKA. Twenty‐four patients were excluded: thirteen refused to participate and eleven had constrained or hinged implants. The study population consisted of 26 men (38.2%) and 42 women (61.8%) with a mean age of 71.2 ± 5.8 years (range: 53–85) and a mean BMI of 28.7 ± 7 (range: 18.4–47.7). The mean hip‐knee‐ankle angle was 175.7 ± 4.5° (range: 168.3°–190.1°), with a global varus deformity (>3°) observed in 20 cases (29.4%).

### Laxity measurement protocol

**Figure 1:**
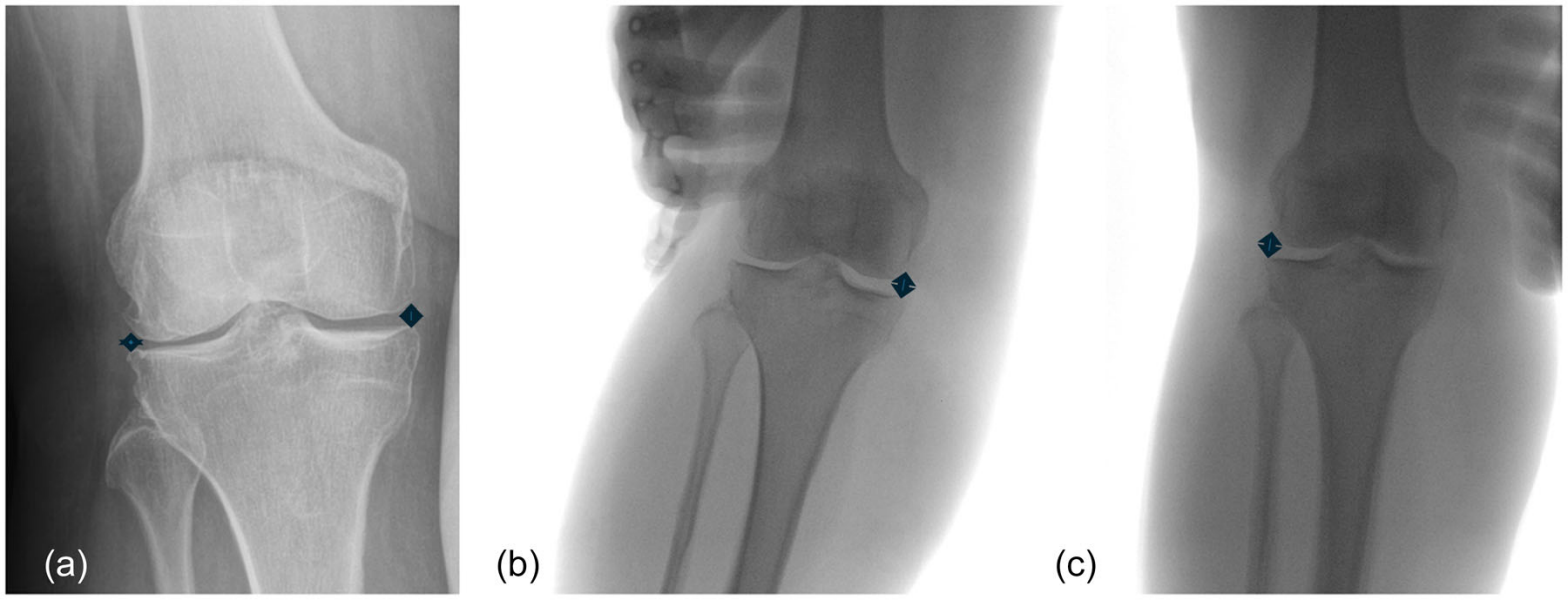
Stress radiographs. (a) Neutral view. (b) Forced valgus for medial compartment. (c) Forced varus for lateral compartment.

Medial and lateral ligamentous laxities were assessed using both ultrasound and radiographic measurements by two independent evaluators (Evaluator 1 and Evaluator 2), blinded to each other's findings, conducted the measurements (Figure 1). In case of discrepancies greater than 1 mm, a third evaluator (Evaluator 3) re‐measured the value.

Dynamic stress manoeuvres were performed manually for both ultrasound and radiographic evaluations, without the use of a stress device used in radiographic evaluations (TELOS) device. Forced varus (to evaluate lateral compartment opening) and valgus (to evaluate medial compartment opening) were applied by the two evaluators in all patients.

Ultrasound laxity measurements were taken at both 0° and 30° of knee flexion. Radiographic stress measurements were only performed at 0°, to limit radiation exposure. The 30° flexion was verified with a goniometer and maintained using physical support.

All measurements were conducted in the same centre according to a standardised protocol validated in a preliminary pilot test on 5 patients. Values were expressed in millimetres (mm).

#### Radiographic measurements

Stress manoeuvres in varus and valgus during radiography required the presence of a person different from the radiographic technician. Four radiographic measurements were taken using standardised anteroposterior calibrated views of the knee in extension (Figure 1):

1. One nonstress image to assess medial and lateral femorotibial compartments.
2. One valgus stress image to assess the medial compartment.
3. One varus stress image to assess the lateral compartment.

The medial compartment was measured between the superomedial aspect of the tibial plateau and the inferomedial femoral condyle. The lateral compartment was measured between the superolateral tibial plateau and the inferolateral femoral condyle.

A metal calibration ball was used for all radiographs to ensure accurate measurement scaling.

After calibration, all measurements were taken manually by two independent investigators (R.H. and J.A.), and the mean of the two values was used for final analysis.

#### Ultrasound manoeuvres

**Figure 2:**
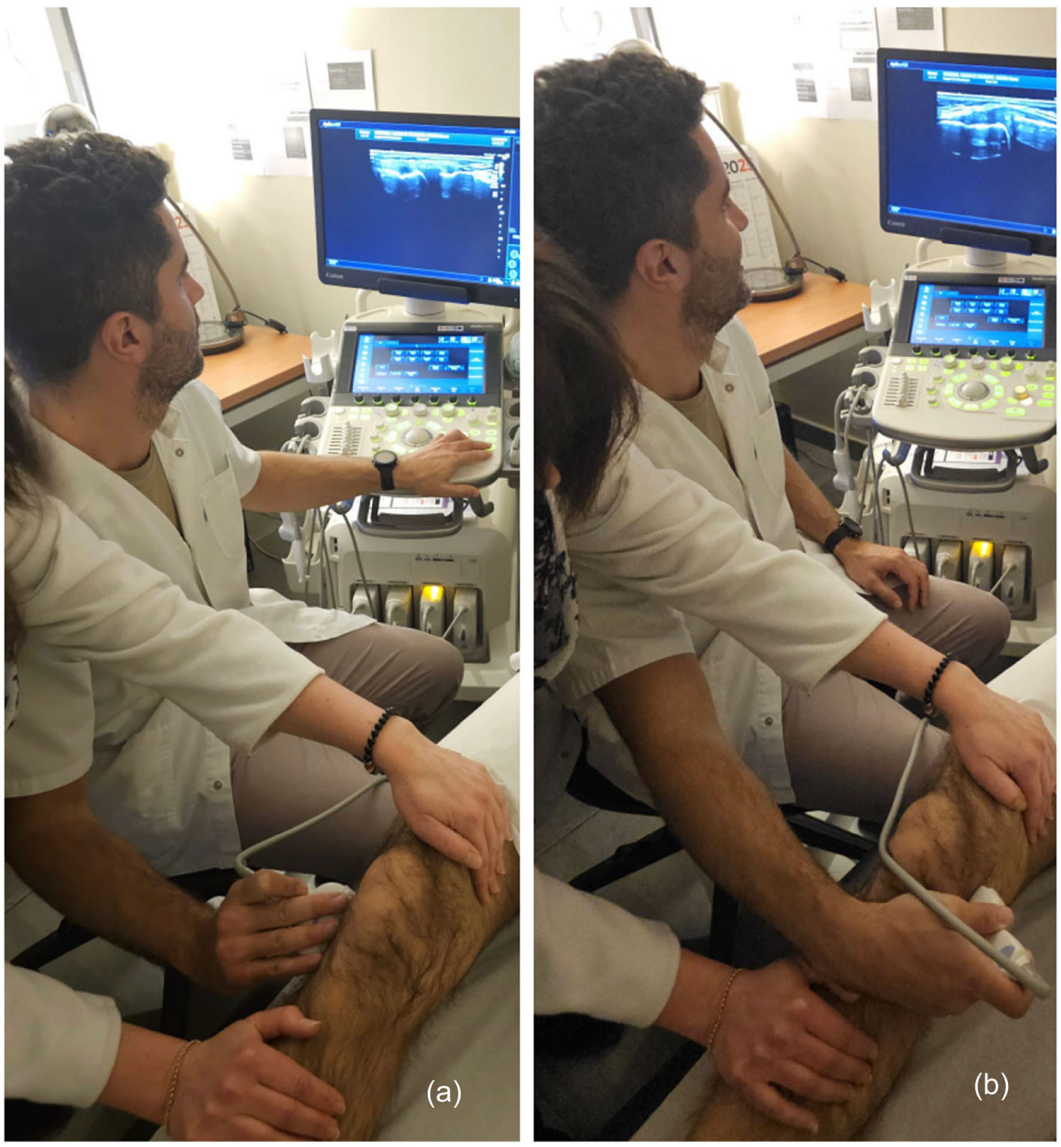
Ultrasound stress testing at 30° of flexion: (a) Lateral compartment in varus stress. (b) Medial compartment in valgus stress.

Stress manoeuvres in varus and valgus required a second person, distinct from the one holding the probe, to apply the stress. Measurements were taken after freezing the reference image (Figure 2).

**Figure 3:**
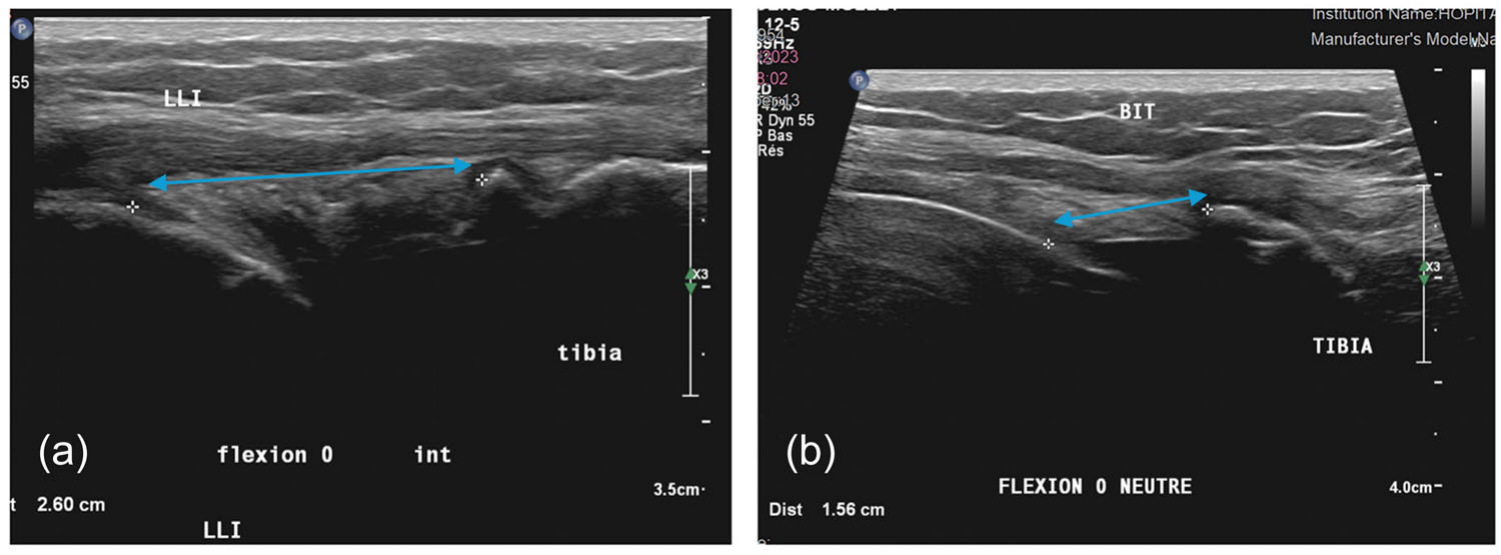
Reference ultrasound landmarks for femorotibial joint space measurement. (a) Typical ultrasound image of the medial joint space. (b) Typical ultrasound image of the lateral joint space. BIT, Iliotibial band; LLI, medial collateral ligament.

Ultrasound landmarks for measuring medial and lateral femorotibial compartments were as follows:

- Medial compartment: The reference image included visualisation of the deep medial collateral ligament and its femoral insertion, producing the characteristic ‘double camel hump’ sign. The medial femorotibial joint space was measured between the superomedial tibial plateau and the inferomedial femoral condyle (Figure 3a).
- Lateral compartment: The probe first located the popliteus tendon, then the lateral condyle proximally, and the iliotibial band and tibia distally as reference landmarks. The joint space was measured between the superolateral tibial plateau and the inferolateral aspect of the lateral femoral condyle (Figure 3b).

These same anatomical landmarks were used regardless of the presence of osteoarthritis or TKA. In cases with osteophytes, measurements were taken from the native bony margins, excluding osteophytes. In cases of prosthetic overhang, measurements were still possible using visible bony edges.

### Outcome measures

Ultrasound laxity measurements were taken at both 0° and 30° of flexion. Values were expressed in millimetres (mm), calculated as the difference in joint space (medial or lateral) between stress and neutral positions.

- Lateral laxity was defined as the difference between joint space under varus stress and in the neutral position (Δ varus–neutral).
- Medial laxity was defined as the difference between joint space under valgus stress and in the neutral position (Δ valgus–neutral).

These ultrasound laxity values were recorded for all patients, and comparisons were made with radiographic measurements taken at 0°.

A subgroup analysis was also performed between the osteoarthritis and TKA groups.

### Statistical analysis

Statistical analyses were performed using Medistica (*p*‐value.io), an R‐based medical statistics platform. Data normality was assessed via the Shapiro–Wilk test. Paired *t*‐tests (or Wilcoxon signed‐rank tests in case of nonnormality) were used for continuous variables; chi‐square or Fisher's exact tests were used for categorical data.

Equivalence testing was performed using two one‐sided tests (TOST) with a predefined margin of ±2 mm and bland‐Altman agreement analysis. A minimum sample size of 46 was calculated to achieve 80% power (*α* = 0.05). Paired *t*‐tests (or Wilcoxon signed‐rank test in case of nonnormality.

An interobserver reliability analysis using intraclass correlation coefficients (ICC) demonstrated good (ICC > 0.80) to excellent (ICC > 0.90) agreement across all laxity measurements (Table 1).

**Table 1:** Interobserver reliability of laxity measurements (ICC values).

Modality	Measurement	Knee position (°flexion)	ICC	95% CI
Ultrasound	Medial compartment	0	0.92	0.86–0.96
	Medial compartment	30	0.89	0.81–0.94
	Lateral compartment	0°	0.91	0.84–0.95
	Lateral compartment	30	0.87	0.79–0.93
Radiography	Medial compartment	0	0.88	0.80–0.93
	Lateral compartment	0	0.85	0.76–0.91

Abbreviations: CI, confidence interval; ICC, intraclass correlation coefficients.

## RESULTS

### Ultrasound measurements of peripheral laxities

The mean lateral laxity measured under ultrasound was 2.9 ± 1.3 mm at 0° of flexion (varus–neutral, *p* < 0.001) and 3.9 ± 1.5 mm at 30° of flexion (varus–neutral, *p* < 0.001).

The mean medial laxity was 2.4 ± 1.1 mm at 0° of flexion (valgus–neutral, *p* < 0.001) and 3.0 ± 1.4 mm at 30° of flexion (valgus–neutral, *p* < 0.001). The absolute values are presented in Table 2.

**Table 2:** Ultrasound measurements of medial and lateral joint space.

	0° (*n* = 68)	30° (*n* = 68)
Medial space without stress (mm)	10.6 (±4.5)	10.9 (±4.2)
Medial space in valgus (mm)	13.0 (±4.9)	14.0 (±5.1)
Lateral space without stress (mm)	11.8 (±4.5)	11.6 (±4.0)
Lateral space in varus (mm)	14.8 (±5.2)	15.5 (±5.9)

*Note*: The results are expressed as the mean and standard deviation.

### Comparison between radiographic and ultrasound laxities in extension

No statistically significant differences were observed between ultrasound and radiographic measurements of peripheral knee laxity in extension, for either lateral (*p* = 0.91) or medial (*p* = 0.78) compartments. Results are detailed in Table 3, showing comparable values between the two techniques.

**Table 3:** Differences between ultrasound and radiographic laxity measurements.

Laxity type	Ultrasound at 0°	Radiograph at 0°	Mean difference (Δ)	*p* value
Lateral laxity	2.9 (±1.7)	2.9 (±2.3)	0.04	0.91
Medial laxity	2.4 (±1.6)	2.4 (±2.2)	0.08	0.78

### Correlation between radiographic and ultrasound measurements

A moderate positive correlation was observed between peripheral laxity measurements obtained via radiography and ultrasound. The strength of this correlation varied based on the type of laxity and the knee position (extension or 30° flexion).

At 0° extension, the correlation was stronger for unstressed medial laxity (*r* = 0.66; 95% confidence interval [CI] = 0.49–0.77; *p* < 0.001) than for unstressed lateral laxity (*r* = 0.51; 95% CI = 0.31–0.67; *p* < 0.001).

Under stress, the correlation coefficient for medial valgus laxity was 0.58 (95% CI = 0.39–0.72; *p* < 0.001), and for lateral varus laxity, it was slightly lower at 0.55 (95% CI = 0.36–0.70; *p* < 0.001).

When comparing radiographic measurements at 0° and ultrasound at 30°, the overall correlation remained moderate (*r* = 0.64; 95% CI = 0.48–0.76; *p* < 0.001). This was particularly evident for unstressed medial laxity, with *r* = 0.66 at 0° and *r* = 0.65 at 30° flexion. Full details are provided in Table 4.

**Table 4:** Correlation between ultrasound and radiographic peripheral laxity measurements.

Laxity type	Correlation at 0°	Correlation at 30°	*p* value
Lateral laxity (no stress); *R*(CI)	0.51 (0.31–0.67)	0.54 (0.35–0.69)	<0.001
Medial laxity (no stress); *R*(CI)	0.66 (0.5–0.77)	0.65 (0.49–0.78)	<0.001

Abbreviations: 95% CI, 95% confidence interval; *R*, correlation coefficient.

### Differences between TKA and osteoarthritis subgroups

Subgroup analysis showed that both medial and lateral laxities were measurable at 0° and 30° flexion via ultrasound in both the osteoarthritis and TKA groups. However, a significantly greater lateral ultrasound laxity at 30° flexion was observed in the TKA group (Δ = 2.1 mm, *p* < 0.01), while no differences were noted for the other parameters. All subgroup comparisons are summarised in Table 5.

**Table 5:** Comparison of laxities between total knee arthroplasty (TKA) and osteoarthritis (OA) subgroups (in mm).

Measurement	TKA (*n* = 36)	OA (*n* = 32)	Δ	*p* value
Lateral ultrasound (US) laxity at 0°	3.2 ± 1.9	2.7 ± 1.4	0.5	0.18
Lateral US laxity at 30°	4.9 ± 3.6	2.8 ± 1.7	2.1	<0.01
Medial US laxity at 0°	2.5 ± 1.9	2.4 ± 1.1	0.1	0.84
Medial US laxity at 30°	3.0 ± 2.3	3.1 ± 2.2	0.1	0.84
Lateral X‐ray laxity at 0°	3.9 ± 2.6	1.8 ± 1.2	2.1	<0.001
Medial X‐ray laxity at 0°	3.2 ± 2.5	1.4 ± 1.0	1.8	<0.001

## DISCUSSION

The results of this study show that ultrasound is a reliable method for measuring peripheral knee laxities, demonstrating a moderate correlation with radiographic measurements. These findings support the use of ultrasound as an alternative to conventional methods for evaluating coronal ligament laxity of the knee.

In contrast to the study by Slane et al. [21], conducted on eight cadaveric specimens and reporting no correlation between ultrasound and radiographic measurements of the medial compartment, our larger in vivo study demonstrates a more consistent relationship between the two modalities both for medial and lateral compartments.

Ultrasound measurements in our study revealed average unstressed joint space values of 10.6 mm (medial) and 11.8 mm (lateral) at 0° flexion, and 10.9 mm (medial) and 11.6 mm (lateral) at 30° flexion. Under stress, these values increased significantly in both valgus and varus directions, illustrating the modality's ability to quantify differential laxity.

Moreover, the mean differences between ultrasound and radiographic laxity measurements were minimal and statistically nonsignificant: 0.04 mm for lateral laxity (*p* = 0.91) and 0.08 mm for medial laxity (*p* = 0.78) (Table 3), suggesting that ultrasound is a trustworthy alternative to the radiographic gold standard.

While ultrasound has already been widely used to assess medial laxity following combined anterior cruciate ligament (ACL) and medial collateral ligament (MCL) injuries [2, 13, 14], or to evaluate meniscal extrusion [10], its application in the context of osteoarthritis and TKA is novel. Our findings support this innovative approach. The study by Lutz et al. [13] further confirms that ultrasound can detect laxity differences based on sex and age, demonstrating its sensitivity.

Our results showed a significant correlation between ultrasound and radiographic measurements of both medial and lateral laxities, with coefficients ranging from 0.51 to 0.65 (*p* < 0.001), depending on the type of stress (Table 4). This consistency highlights the accuracy of ultrasound in comparison to standard radiographic techniques.

Furthermore, we observed significant differences between the osteoarthritis and TKA subgroups, particularly in lateral ultrasound laxity at 30° of flexion, which was greater in the TKA group (Δ = −4.8 vs. −2.8 mm, *p* < 0.01; Table 5). This suggests that posterior‐stabilised TKA primarily alters lateral knee balance in flexion.

This study has several limitations that must be acknowledged. First, the relatively small sample size limits the statistical power; as a feasibility study, it did not allow for multivariate analyses within subgroups (TKA vs. osteoarthritis), restricting conclusions about factors influencing joint laxity. Additionally, selection bias is possible, as some patients declined participation, which could limit generalisability. Third, the lack of strict standardisation in stress manoeuvres, especially at 30° flexion, may affect measurement reproducibility. Finally, manual stress application in both radiographic and ultrasound evaluations may limit the standardisation of the measurements and partly account for the variability in correlation coefficients observed. The Telos® device offers a standardised application of stress, which can improve the reproducibility of measurements. However, some studies have shown better inter‐observer reliability with manual stress [9, 11, 15], supporting our choice of the manual technique for its clinical feasibility and accessibility.

Despite these limitations, ultrasound represents a compelling alternative to dynamic radiographs for evaluating joint laxity due to its lack of radiation, real‐time dynamic capability at various flexion angles, and high accessibility in both outpatient and postoperative settings.

A promising perspective would be the integration of ultrasound in the intraoperative setting to refine ligament balance assessment. This could be an alternative where navigation systems or ligament balancers [18, 19] are unavailable, providing a more precise dynamic analysis that reflects the patient's actual joint behaviour [16]. Such an approach may help replicate preoperative laxity profiles and reduce the risk of postoperative instability. De Saint Vincent et al. [20] have shown that postoperative lift‐off observed on standard radiographs can indicate residual laxity and potential secondary instability. Implementing pre‐ and intraoperative ultrasound assessment could help anticipate and prevent this complication.

Additionally, the study by Lutz et al. [13] confirmed that ultrasound reliably evaluates knee stability after acute combined ACL and MCL injuries, reinforcing its legitimacy for dynamic laxity assessments. Finally, recent studies [17, 22] have demonstrated that constrained and semiconstrained prostheses yield similar outcomes in revision surgery for instability—one of the main indications for reoperation. Improved intraoperative assessment of laxity could therefore help reduce the rate of revision procedures for postoperative instability.

From a health‐economic perspective, replacing dynamic stress radiographs with ultrasound yields tangible savings in both time and direct costs. In our cohort, a complete bilateral ultrasound assessment took about 40% less time than the radiographic protocol (which includes positioning, exposure and calibration). When ultrasound is performed immediately during the clinical consultation—without being billed as a separate technical act—it generates no additional fee, whereas stress radiography requires a dedicated imaging session and a reimbursed act.

Even if ultrasound were invoiced independently, its tariff for a standard musculoskeletal scan remains within the same price bracket as radiography while still eliminating radiation exposure, freeing radiology‐suite time and avoiding consumables (cassette covers, calibration spheres). Recent health‐technology‐assessment reports (2023–2024) converge in recommending nonirradiating imaging whenever diagnostic performance is equivalent. Taken together, the shorter examination time, minimal consumable cost and absence of ionising radiation strongly support ultrasound as a cost‐effective first‐line tool for assessing knee laxity.

## CONCLUSION

Stress ultrasound is a reliable tool for analysing medial and lateral peripheral knee laxities in both osteoarthritic knees and after TKA. While it could requires trained personnel, this technique represents a viable alternative to conventional stress radiographs.

## AUTHOR CONTRIBUTIONS

Ammar Ghabi, Lilia Gharbi, Robin Hattenberger and Jérôme Desmolliens performed the dynamic manoeuvres. Ammar Ghabi wrote the initial manuscript. Jean‐François Gonzalez, Grégoire Micicoi and Bernard de Geofroy performed the surgery and corrected the different versions of the draft. All the authors approved the submitted version.

## CONFLICT OF INTEREST STATEMENT

Jean‐François Gonzalez is a consultant for Amplitude. The remaining authors declare no conflicts of interest.

## ETHICS STATEMENT

This was a retrospective bicentric study conducted on a consecutive series of patients followed in outpatient clinics between 1 October 2023, and 31 January 2024. The study was conducted in accordance with the Declaration of Helsinki and approved by the institutional review board (IORG0012275—iULS‐University Institute for Locomotion and Sport. IRB00014528 ULS—University Institute for Locomotion and Sports). All patients participated voluntarily and gave written informed consent.

## Data Availability

The data that support the findings of this study are available on request from the corresponding author. The data are not publicly available due to privacy or ethical restrictions.
